# Activation of a Silent Polyketide Synthase SlPKS4 Encoding the C_7_-Methylated Isocoumarin in a Marine-Derived Fungus *Simplicillium lamellicola* HDN13-430

**DOI:** 10.3390/md21090490

**Published:** 2023-09-13

**Authors:** Jing Yu, Xiaolin Liu, Chuanteng Ma, Chen Li, Yuhan Zhang, Qian Che, Guojian Zhang, Tianjiao Zhu, Dehai Li

**Affiliations:** 1 Key Laboratory of Marine Drugs, Chinese Ministry of Education, School of Medicine and Pharmacy, Ocean University of China, Qingdao 266003, China; jingjingyu95@163.com (J.Y.); 17865327317@163.com (X.L.); ma_chuanteng@163.com (C.M.); 17629508296@163.com (C.L.); cheqian064@ouc.edu.cn (Q.C.); zhangguojian@ouc.edu.cn (G.Z.); 2 School of Pharmaceutical Science, Shandong University, Jinan 250100, China; 202100260059@mail.sdu.edu.cn; 3 Laboratory for Marine Drugs and Bioproducts, Pilot National Laboratory for Marine Science and Technology (Qingdao), Qingdao 266237, China

**Keywords:** isocoumarins, nonreducing polyketide synthase (nrPKS), *Simplicillium lamellicola*, genome mining, silent gene clusters

## Abstract

Coumarins, isocoumarins and their derivatives are polyketides abundant in fungal metabolites. Although they were first discovered over 50 years ago, the biosynthetic process is still not entirely understood. Herein, we report the activation of a silent nonreducing polyketide synthase that encodes a C_7_-methylated isocoumarin, similanpyrone B (**1**), in a marine-derived fungus *Simplicillium lamellicola* HDN13-430 by heterologous expression. Feeding studies revealed the host enzymes can change **1** into its hydroxylated derivatives pestapyrone A (**2**). Compounds **1** and **2** showed moderate radical scavenging activities with ED_50_ values of 67.4 µM and 104.2 µM. Our discovery fills the gap in the enzymatic elucidation of naturally occurring C_7_-methylated isocoumarin derivatives.

## 1. Introduction

Fungi have proven to be a tremendous source of new bioactive lead compounds with thousands of bioactive compounds isolated [1,2]. Meanwhile, whole-genome sequencing data revealed that the number of biosynthetic gene clusters encoded in fungi is much larger than the types of natural products isolated, which indicates that a major portion of biosynthetic gene clusters are still silent or poorly expressed [3,4]. To activate these silent gene clusters and increase the silent metabolic potential, a variety of techniques have been developed, including epigenetics regulation, co-culture, precursor feeding, heterologous expression, changing fermentation parameters and ribosome engineering, etc. [5,6].

Among these, heterologous expression has unique advantages, especially to achieve the de novo biosynthesis of compounds in a heterologous host, which benefits from genetic tractability, short life-cycle, and high bio-safety [7]. As for the activation of silent gene clusters in fungi, heterologous expression shows special superiority, such as: (1) it is more controllable compared to other activation methods, especially, the activation is orientated instead of randomly; (2) the ideal chassis cells with simple metabolite backgrounds make it easy to perform the isolation of targeted compounds; (3) it is still effective without regulators, selective markers, or strain genetic operating system, which is frequently the major obstacle in non-model fungi [8,9,10]. 

**Figure 1:**
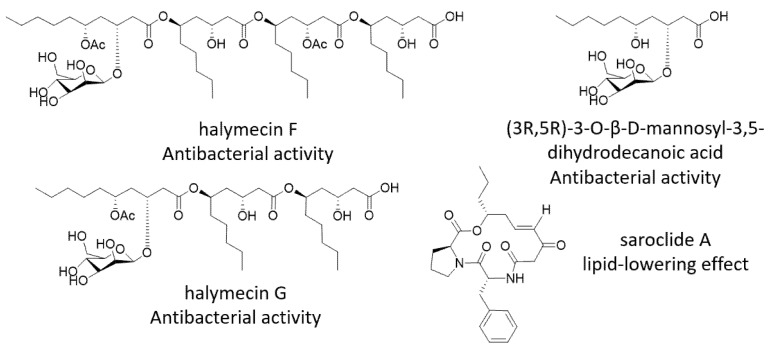
Bioactive compounds reported from *S. lamellicola*.

The fungal specie, *Simplicillium lamellicola*, has great ecological and commercial importance due to the exceptional bioactivities, particularly in microbial biopesticide [11,12,13,14]. However, to our best knowledge, four new compounds, reported in the specie (Figure 1), indicate a great potential for bioactive secondary compounds from *S. lamellicola*. Isolated from a marine sediment collected in Pritz Bay, *S. lamellicola* HDN13-430 was the first strain of this specie isolated from Antactica, which made the genome mining worthwhile. During our ongoing genome mining work on the fungal strain *S. lamellicola* HDN13-430, a nonreducing polyketide synthase (nrPKS), termed SlPKS4, attracts our attention. SIPKS4 showed a low sequence identity to other PKSs. With the expression test of cDNA, the nrPKS, together with the gene cluster, were proven to be completely silent under regular laboratory conditions (Figure S1). Due to the lack of regulators in the native strain, the following heterologous expression of SlPKS4 in *Aspergillus nidulans* lead to the yield of two isocoumarin derivatives: similanpyrone B (**1**) and pestapyrone A (**2**). Compounds **1** and **2** showed radical scavenging activities, while no activity in the antibacterial bioassays was observed. Although compounds **1** and **2** were first described more than 50 years ago, the biosynthetic process is still not entirely understood. We proposed the biosynthetic pathway of compounds **1** and **2**, and conducted phylogenetic analysis with other PKSs responsible for the synthesis of isocoumarin derivatives. 

## 2. Results

### 2.1. Bioinformatic Analysis of the Target nrPKS and the Gene Cluster in S. lamellicola HDN13-430

**Figure 2:**
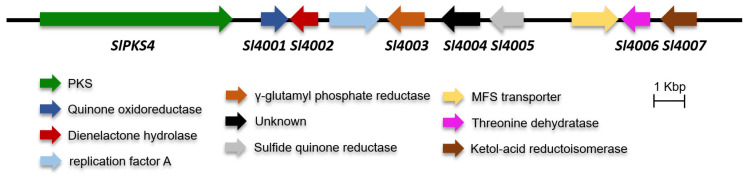
Organization and proposed function of *SlPKS4* and tailoring genes.

To probe the metabolic potential of *S. lamellicola* HDN13-430, the whole-genome sequencing and analysis were performed. The prediction of secondary metabolites using antiSMASH indicated 12 PKSs, 16 NRPSs, 3 terpenes, 6 hybrids and 2 other types biosynthetic gene clusters (Figure S2). During analyzing the PKSs of strain *S. lamellicola* HDN13-430, an nrPKS which we termed as *SlPKS4* (Figure 2), exhibits low identities with known nrPKSs, while the highest similarities of 41.46% and 41.20% at the amino acid level were donated by pkbA [15] and andM [16], which are responsible for the biosynthesis of compounds 3-methylorsellinic and 3,5-dimethylorsellinic acid, respectively. The low-similarity PKS *SlPKS4*, located in a cluster with seven tailoring enzymes termed Sl4001-Sl4007, with proposed functionsas quinone oxidoreductase, dienelactone hydrolase, γ-glutamyl phosphate reductase, hypothetical protein, sulfide quinone reductase, threonine dehydratase, ketol-acid reductoisomerase, respectively (details in Table S2). The proposed functions of enzymes located in the cluster are all uncommon with a rare report in secondary metabolites biosynthesis. However, further analysis of gene transcription status by RT-PCR of six media based on OSMAC (one strain many compounds) and epigenetic regulation strategies, shows that the cluster including *SlPKS4* is totally silent under regular laboratory culture conditions (Figure S1). Also, there are no reports about the construction of a genetic operating system on the specie *S. lamellicola*, which prompts us to investigate the function by heterologous biosynthesis in *A. nidulans*. 

### 2.2. Heterologous Expression of the Gene Cluster and Elucidation of Compounds ***1*** and ***2***

**Figure 3:**
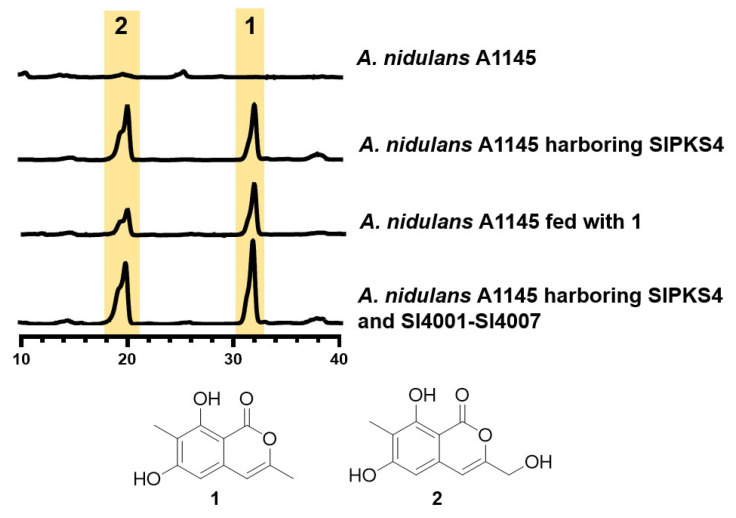
HPLC analysis of the secondary metabolites in *A. nidulans* strains. UV absorptions at 280 nm are illustrated. HPLC full chromatogram of the original *A. nidulans* and the strain harboring SlPKS4 were provided to prevent the presence of compound **2** in original *A.nidulans* (Figure S5). HPLC analysis method: 5:95 to100:0 MeOH-H_2_O (with 0.1% trifluoroacetic acid), 40 min, 1 mL/min.

To demonstrate the function of SlPKS4, a 9547 bp fragment containing the whole genomic sequence of SlPKS4, plus a downstream region of 526 bp containing the native terminator, were amplified via PCR and cloned into the expression vector pANU-*SlPKS4* by homologous recombination in *Saccharomyces cerevisiae*. The obtained construct, pANU-*SlPKS4*, was introduced into *A. nidulans* A1145 by polyethylene glycol (PEG)-mediated protoplast transformation. Integration transformants, including pANU-*SlPKS4*, were grown on liquid CD-Starch medium following selection by uridine and uracil autotrophy and subsequent confirmation by PCR amplification [17]. The cultures were extracted with ethyl acetate and analyzed by LC-MS for secondary metabolites. As shown in Figure 3, two additional peaks of compounds **1** and **2** were detected in the extract compared to the control strain containing the empty vector. The two compounds share similar UV spectra with absorption maxima at 240, 280 and 330 nm, together with [M + H]^+^ ions at *m/z* = 207.1 and 223.2, respectively, indicating similar structures and differences coming from hydroxylation (Figure S2). Following large-scale fermentation, isolation and structural elucidation by 1D NMR analysis (Tables S3 and S4 and Figures S8–S11) confirmed compounds **1** and **2** to be similanpyrone B and pestapyrone A, respectively [17,18]. The literature review concludes that both compounds belong to the group of isocoumarins. 

Similarly, the tailoring enzymes Sl4001-Sl4007 were separately cloned, constructed on expression plasmids and introduced into *A. nidulans* A1145 (Figure S4). Unexpectedly, after 4 days of culturing followed by extraction with ethyl acetate, no new compound, except **1** and **2**, was detected by LC-MS analysis (Figure 3). Double checking the gene transcription status by RT-PCR was performed, confirming that all seven tailoring enzymes, together with SlPKS4, were expressed properly (Figure S6), which exclude the possibility of unexpression. 

Compound **1** has undergone investigation through chemical synthesis [19] and isotope labeling [20,21] since the 1980s, however, the enzyme responsible for the biosynthesis has not been reported until now. Our report about SlPKS4 is the first discovery of PKS responsible for compound **1**. Meanwhile, isocoumarins derivatives were generally discovered from the fungal genera *Penicillium*, *Ceratocystis*, *Fusarium*, *Artemisia*, *Aspergillus*, *Cladosporium*, *Oospora*, and *Hydrangea* [22]. To our best knowledge, there is no coumarins or isocoumarins reported from the fungal genus *Simplicillium,* so this is also the first time to prove that the fungal genus *Simplicillium* has the ability to produce isocoumarin derivatives.

### 2.3. Origin Verification of Compound ***2*** by Biotransformation Assay

**Figure 4:**
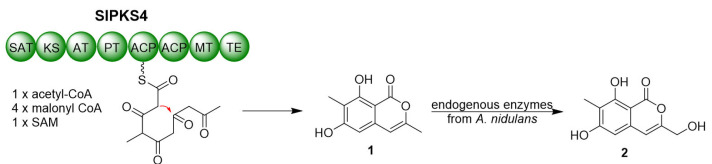
Biosynthetic pathway of **1** and **2** in *A. nidulans* A1145.

Unexpected accumulation of compound **2** as a hydroxylated derivative of **1** raises the question on the origin of the hydroxylation activity, due to no corresponding enzymes for the related hydroxylation reaction. Inspired by a previous work by Li et al. [23], we postulated that endogenous enzymes from *A. nidulans* were also in charge of the conversion of **1** to **2**. Intrigued by this hypothesis, we conducted a feeding experiment of compound **1** in *A. nidulans* A1145. After four days feeding with compound **1**, compound **2** was clearly present in the culture extract after LC-MS analysis (Figure 3). This proved that *A. nidulans* can modify the initial polyketide product **1** by hydroxylation at the methyl (Figure 4). Unfortunately, no *A. nidulans* candidate enzymes could be anticipated for the process. 

### 2.4. Bioactivities of Compounds ***1*** and ***2***

In previous reports, compound **1** was tested for cytotoxic activities against the human cancer cell lines Hela, A549, HepG2 and the mouse lymphoma cell line L5178Y, antimicrobial activity against Gram positive (*Staphylococcus aureus* ATCC 25923 and *Bacillus subtilis* ATCC 6633) and Gram negative (*Escherichia coli* ATCC 25922 and *Pseudomonas aeruginosa* ATCC 27853) bacteria, *Candida albicans* ATCC 10231, and multidrug-resistant isolates from the environment [17,18,24], while compound **2** was evaluated for the cytotoxicity activities against a panel of cancer cell lines (A549, HL-60, K562 and L5178Y) [17,25]. All the above investigations proved to be inactive. In our research, the antimicrobial activities against MRCNS (Methicillin-Resistant Coagulase-Negative *Staphylococci*), MRSA (Methicillin-Resistant *Staphylococcus Aureus*), *S. aureus*, *Acinetobacter baumannii*, *B. cereus*, *B. subtilis*, *P. aeruginosa*, and *C. albicans* were evaluated, but none of them presented an antimicrobial effect under the concentration of 30 µM. Meanwhile, the radical scavenging assay based on 1,1-diphenyl-2-picrylhydrazyl radical 2,2-diphenyl-1-(2,4,6-trinitrophenyl) hydrazyl (DPPH) was used to test the radical scavenging activity of compounds **1** and **2**, and they showed similar effects with ED_50_ values of 67.4 µM and 104.2 µM (the value of ascorbic acid was 12.6 µM as positive control).

## 3. Discussion

Coumarins and isocoumarins are a large family of lactonic natural products, abundant in various organisms including bacteria, fungi, lichens, liverworts, sponges, plants and insects [22,26]. The widespread distribution and possession of a broad spectrum of pharmacological activities, including antifungal, anti-inflammatory, cytotoxic, and antimicrobial properties, have led to the continuous discovery of novel isocoumarin compounds [22,27,28]. Structurally, there are six chemical active positions (C_3_ to C_8_) of isocoumarin, which make a substantial contribution to the formation of diverse chemical derivatives containing alkyl, halogen, heterocyclic, aryl, etc. Isocoumarins have garnered significant attention in total synthesis due to their utility as key intermediates in the production of valuable compounds, such as isoquinolines and isochromenes [27,29,30]. These diverse chemical substitution patterns significantly augment the structural complexity and broaden the range of biological and pharmacological activities of isocoumarins [31,32,33]. 

Several biosynthetic gene and gene clusters responsible for coumarins and isocoumarins have been discovered [23,32,33,34,35]. In fungi, isocoumarin derivatives are primarily derived from the polyketide pathways, which are typically catalyzed by nonreducing polyketide synthase (nrPKS), containing domains such as starter unit ACP transacylase (SAT), β-ketoacyl synthase (KS), acyl transferase (AT), product template (PT), acyl carrier protein (ACP), methyltransferase (MT), and thioesterase (TE) [22,23,32]. However, despite recent significant advancements in the chemical synthesis of isocoumarins, there are relatively few PKSs responsible for their biosynthesis that have been documented, compared to the abundance of isocoumarin derivatives. In fungi, the elucidated biosynthetic PKSs could be divided into two categories. The first class, represented by Pcr9304 and cla3, have no MT domain, which corresponds with the absence of methyls on C_3,5,7_, while only one report of the second class exists with C_3,5_-dimethyl directed by AcreC. To date, there is no biosynthetic enzyme report about C_7_-methyl isocoumarin derivatives. 

**Figure 5:**
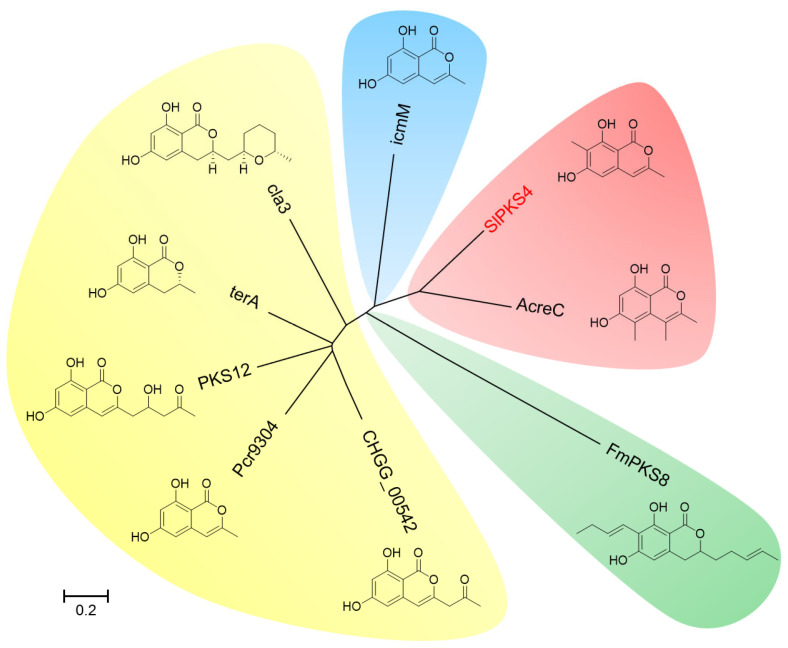
Phylogenetic tree analysis of SlPKS4 and reported PKSs synthesizing isocoumarin derivatives.

Inspired by the uncommon carbon substituent at C_7_-methyl of **1** and **2** with raising reported activities [22,26], we performed a comparison towards reported PKSs synthesizing isocoumarin derivatives by phylogenetic analysis (Figure 5). The results demonstrated that SlPKS4 is close to AcreC, which also contains an MT domain compared to others. NCBI BLASTP at the amino acid level between SlPKS4 and AcreC shows a sequence identity of 36.89%. However, the reason why the MT domain directed SAM on different sites remains unknown. 

Despite being located in a cluster, the *SlPKS4* gene is sufficient for compound **1** production. The reasons why the tailoring enzymes were ineffective are still unknown. We proposed that it is possibly because of the inactivation of the genes encoding tailoring enzymes during the rearrangements and breakages, or as a result of being flanked by potential transposons [36,37,38]. Another hypothesis was that the post-translational modification rules may differ among different host strains, lead to wrong enzyme structures, and ultimately became inactive. Moreover, we did comparative analysis between the gene cluster containing SlPKS4 and other biosynthetic gene clusters producing isocoumarin using clinker (Figure S7). The results suggested that our gene cluster was low and/or similar as a whole, and the tailoring enzymes were least conversed.

## 4. Materials and Methods

### 4.1. General Experimental Procedures

Genomic DNA samples were prepared using the CTAB isolation buffer at pH 8.0 (20 g/L cetyltrimethylammonium bromide, 1.4 M sodium chloride, and 20 mM EDTA). Polymerase chain reaction (PCR) was performed using Phusion^®^ High-Fidelity DNA Polymerase (New England Biolabs, NEB, Beijing, China). PCR analyses were conducted using a 2×Hieff^®^ PCR Master Mix (With Dye, Yeasen, Shanghai, China). DNA restriction enzymes were used as recommended by the manufacturer (New England Biolabs, NEB, Beijing, China). RT-PCR analysis was performed using Direct-zol™ RNA MiniPrep (Zymo Research, Irvine, CA, USA) and PrimeScript RT-PCR Kit (TaKaRa, Gunma, Japan). The gene-specific primers are listed in Table S1. Custom oligonucleotides synthesis and fragments sequencing were served by Shanghai Sangon DNA Technologies. LC-MS was performed using an Acquity UPLC H-Class coupled to a SQ Detector 2 mass spectrometer using a BEH C18 column (1.7 μm, 2.1 × 50 mm, 1 mL/min) (Waters Corporation, Milford, MA, USA). Semi-preparative HPLC (YMC Co., Ltd., Kyoto, Japan) was performed on an ODS column (YMC-Pack ODS-A, 10 × 250 mm, 5 μm, 3 mL/min). ^1^H NMR and ^13^C NMR spectra were recorded on an Agilent 500 MHz DD2 spectrometer (Agilent Technologies Inc., Santa Clara, CA, USA). The spectra were processed by the software MestReNova 6.1.0 (Metrelab, Coruña, Spain). NMR data are provided in Tables S3 and S4, and spectra in Figures S8–S11.

### 4.2. Materials and Culture Conditions

The fungal strain *S. lamellicola* HDN13-430 was isolated from marine sediment collected in Antarctic Pritz Bay. The strain was identified by an internal transcribed spacer (ITS) sequence and the sequence data were submitted to GenBank (GenBank accession number KY794926). The strains were deposited at the Ministry of Education of China, School of Medicine and Pharmacy, Ocean University of China, Qingdao, China. 

For RNA isolation, *S. lamellicola* HDN13-430 was cultured at 28 °C on media potato dextrose agar (PDA, 20% potato, 2% dextrose, and 2% agar) plates for 5 days, and inoculated into Elenmeyer flasks containing 150 mL of 6 different liquid culture medium for 4 days. For genomic DNA extraction, *S. lamellicola* HDN13-430 was cultured at 28 °C on PDA plates for 7 days.

*Escherichia coli* strain XL-1 was used for plasmids preservation and amplification. *Saccharomyces cerevisiae* Y31 was used for in vivo DNA recombination for plasmids construction. 

*A. nidulans* A1145 was grown at 28 °C in CD (0.1% Glucose, 0.5 *v*/*v*% 20×Nitrate salts, 0.01 *v*/*v*% Trace elements, and 2% agar for solid media) media for sporulation, CDS (0.1% Glucose, 1.2 M D-sorbitol, 0.5 *v*/*v*% 20×Nitrate salts, 0.01 *v*/*v*% Trace elements, and 2% agar for solid media) to screen transformants or in CD-ST (2% starch, 2% Casamino acids, 5 *v*/*v*% 20×Nitrate salts, 0.1 *v*/*v*% Trace elements) media for heterologous expression and compound production. All medias were prepared with appropriate supplements, including 10 mM uridine, 5 mM uracil and/or 0.5 µg/mL pyridoxine HCl and/or 2.5 µg/mL riboflavin, depending on the plasmids being transformed.

### 4.3. Sequence Analysis of the SlPKS4 Gene

The whole genome sequencing data was analyzed by antiSMASH [39]. The phylogenetic analysis was conducted with the MEGA7 software [40], with the amino acid sequences of SlPKS4 and reported PKSs synthesizing isocoumarin derivatives retrieved from National Center for Biotechnology Information (NCBI). The conserved domain of the SlPKS4 protein was scanned by the InterProScan program [41]. Comparative analysis between the gene cluster and other isocoumarin BGCs (Figure S7) was conducted by clinker [42].

### 4.4. Heterologous Expression of SlPKS4 and Sl4001-Sl4007 in A. nidulans A1145

Genes *SlPKS4* and *Sl4001–Sl4007* were amplified from genomic DNA extract from *S. lamellicola* HDN13-430. Plasmids pANU, pANR, pANP with auxotrophic markers for uracil (pyrG), riboflavin (riboB) and pyridoxine (pyroA) were digested with PacI and NotI, PacI and BamHI, PacI and HindIII, respectively, and used as vectors to insert genes. The corresponding heterologous expression plasmids were obtained by in vivo yeast homologous recombination in *S. cerevisiae* Y31. The correct colonies checked by PCR were combined, and subjected to yeast miniprep to obtain the plasmids. The plasmids obtained from yeast miniprep using Plasmid Miniprep Kit (Zymo Research, Irvine, CA, USA) were introduced into the competent cells of *E. coli* XL-1. After plasmid extraction from *E. coli* to obtain transformants with a single plasmid, plasmids were sequenced to confirm identities.

For protoplast formation of *A. nidulans* A1145, the strain *A. nidulans* A1145 was first grown on CD plates at 37 °C for 3 days and fresh spores were collected and stored. Then, the spores were germinated in a 250 mL Erlenmeyer flask containing CD media at 37 °C and 180 rpm for about 8 h. Mycelia were gathered by centrifugation at 4000 rpm for 15 min, and washed by 25 mL osmotic buffer (1.2 M MgSO_4_, 10 mM sodium phosphate, pH 5.8). Subsequently, the mycelia were suspended into 10 mL of osmotic buffer containing 30 mg lysing enzymes from Trichodema harzianum (Sigma) and 20 mg Yatalase (TaKaRa), transferred into an empty sterile bottle, and cultured in a shaker of 28 °C at 80 rpm overnight to form protoplast. After the whole night, the mixture was collected in a 50 mL centrifuge tube and covered gently with isopyknic protoplast trapping buffer (0.6 M sorbitol, 0.1 M pH 7.0 Tris-HCl). After centrifugation at 4000 rpm for 15 min at 4 °C, protoplasts were collected in the interface of the above two buffers. The protoplasts were then transferred to a sterile 50 mL centrifuge tube and washed by 20 mL STC buffer (1.2 M sorbitol, 10 mM CaCl_2_, 10 mM pH 7.5 Tris-HCl). The protoplasts were resuspended in 2 mL STC buffer for transformation.

For protoplast transformation of *A. nidulans* A1145, the necessary plasmids and the corresponding empty plasmids (the desired strains were regarded as control in the following crude analysis) were added to 100 µL *A. nidulans* A1145 protoplast suspension prepared above and the mixture was incubated on ice for 60 min. Next, 600 μL of polyethylene glycol (PEG) solution (60% PEG, 50 mM calcium chloride and 50 mM pH 7.5 Tris-HCl) was added to the protoplast mixture, and the mixture was incubated at room temperature for an additional 25 min. The mixture was spread on the regeneration solid CDS medium with appropriate supplements, including 10 mM uridine, 5 mM uracil and/or 0.5 µg/mL pyridoxine HCl and/or 2.5 µg/mL riboflavin, depending on the plasmids being transformed and incubated at 37 °C for around 3 days.

### 4.5. Fermentation and Extraction

For small-scale analysis, the strains with expression plasmids and the control strains were grown on CD plates with appropriate supplements for 3 days at 37 °C. Shortly after sporing, they were inoculated into 150 mL of CD-ST medium with appropriate supplements and cultured at 28 °C, 180 rpm. Meanwhile, they were spread on solid CD-ST medium with appropriate supplements, respectively. Four days later, the cultures were extracted with ethyl acetate and the organic phase was evaporated and dissolved in MeOH, which was analyzed by HPLC. 

For compound isolation, the selected strain was initially handled the same as above. Then, a large-scale fermentation was performed in 500 mL Erlenmeyer flasks (total 5 L) for further incubation. The broth was extracted three times with ethyl acetate to provide a total of 15 L of extract solution. The organic phase was evaporated under reduced pressure to afford a crude residue (6 g).

### 4.6. Compound Isolation

The extract was applied to MPLC (ODS) using a stepped gradient elution of MeOH-H_2_O (40:60 to 100:0 for 60 min at 10 mL/min) to yield eight subfractions (Fr.1-Fr.8). Fr.4 was separated by semi-preparative HPLC (YMC-pack ODS, 10 × 250 mm, 3.0 mL/min) to afford **2** (65% MeOH in H_2_O, 0.1% THF, 8.5 mg, t_R_ = 25 min) and Fr.5 was purified by semi-preparative HPLC to obtain **1** (72% MeOH in H_2_O, 0.1% THF, 11.0 mg, t_R_ = 24 min). The purity of the compounds was checked by LC-MS and the structures were confirmed by ^1^H and ^13^C NMR spectra.

### 4.7. Biotransformation Assay of ***1*** in A. nidulans

For the biotransformation assay in *A. nidulans*, compound **1** was dissolved in a minimal amout of DMSO and then added into CD-ST liquid media at a final concentration of 100 μM. The strain *A. nidulans* A1145 was inoculated on the prepared medium and grown for 4 days at 28 °C 180 rpm. Meanwhile, a CD-ST medium with equal amount of compound **1** was prepared without any strain, and was cultivated under the same condition as the control. The cultures were then extracted by ethyl acetate and the organic phase was dried, dissolved in methanol and detected by LC-MS.

### 4.8. Assay of Antimicrobial and Antioxidant Activities

Antimicrobial activities of compounds **1** and **2** were tested by the micro broth dilution method, as mentioned in the previous study [43]. The microorganism suspension (198 μL, 106 cfu/mL) in MH medium (Casein Acid Hydrolysate 17.5 g/L, beef extract 2 g/L, soluble starch 1.5 g/L, pH 7.3) was added to each well of 96-well plates. Solutions (6 mM) of the compounds and positive drugs were made up in DMSO and dispensed into 96-well plates to provide 16 concentrations in the range of 30–0.02 μM. Incubated at 28 °C for 9 h (*Candida albicans* in 37 °C for 12 h), the growth inhibition was recorded. 

In the DPPH scavenging assay, samples to be tested were dissolved in MeOH and the solution (160 μL) was dispensed into wells of a 96-well microtiter tray. Forty microliters of the DPPH solution in MeOH were added to each well. The mixture was shaken and left to stand for 30 min. After the reaction, absorbance was measured at 510 nm, and the percent inhibition was calculated. ED_50_ values denoted the concentration of sample required to scavenge 50% of the DPPH free radicals [44].

## 5. Conclusions

In summary, guided by the bioinformatic analysis of *S. lamellicola* HDN13-430, we successfully activated the silent nrPKS gene *SlPKS4* in *A. nidulans*, and proved its function as an isocoumarin synthase. Furthermore, we demonstrated by feeding experiment that **2** are modification products of **1** by uncharacterized endogenous host enzymes. Based on the DPPH scavenging assay, Compounds **1** and **2** showed moderate radical scavenging activities with ED_50_ values of 67.4 µM and 104.2 µM, respectively, which was the first time for this to be measured. Our results provide one additional example that the products of a heterologous expressed gene can be further converted by host enzymes. Furthermore, this is the first report of nrPKS responsible for the biosynthesis of compound **1**. This is also the first report that the fungal specie *S. lamellicola* could produce isocoumarin derivatives, which expands the production methods of isocoumarins compounds.

## Data Availability

The gene sequence of *SlPKS4* was uploaded to GeneBank (Accession Number: OR519897).

## Supplementary Materials

The following supporting information can be downloaded at: https://www.mdpi.com/article/10.3390/md21090490/s1, Table S1: The primers used in this study; Table S2: Analysis of gene cluster; Table S3: NMR data of **1** in DMSO-*d*_6_; Table S4: NMR data of **2** in CD_3_OD; Figure S1: RT-PCR analysis for gene transcription status. The results showed that all seven tailoring enzymes together with SlPKS4 were totally silent under 6 regular laboratory conditions; Figure S2: AntiSMASH analysis results of the genome of the strain *S. lamellicola* HDN13-430; Figure S3: UV absorptions of compounds **1** and **2**; Figure S4: Maps of the vectors pANU-*SlPKS4*, pANU-*SlPKS4*+*Sl4004*, pANR-*Sl4001+5+7* and pANP-*Sl4002+3+6*; Figure S5: HPLC full chromatogram of the original *A. nidulans* and the strain harboring SlPKS4. The results prevent the presence of compound **2** in original *A.nidulans*; Figure S6: RT-PCR analysis for gene transcription status. The results showed that all seven tailoring enzymes together with SlPKS4 were expressed properly; Figure S7: Comparative analysis between the gene cluster and other isocoumarin BGCs by clinker; Figure S8: ^1^H NMR (500 MHz, DMSO-*d*_6_) spectrum of compound **1**; Figure S9: ^13^C NMR (125 MHz, DMSO-*d*_6_) spectrum of compound **1**; Figure S10: ^1^H NMR (500 MHz, CD_3_OD) spectrum of compound **2**; Figure S11: ^13^C NMR (125 MHz, CD_3_OD) spectrum of compound **2**. Gene Sequence of *SlPKS4.*
