# Supplementary material for: Activation of a Silent Polyketide Synthase SlPKS4 Encoding the C7-Methylated Isocoumarin in a Marine-Derived Fungus Simplicillium lamellicola HDN13-430
Source: Mar Drugs. 2023 Sep 13;21(9):490. doi: 10.3390/md21090490 (PMC10532586; doi:10.3390/md21090490)
Supplement: Supplementary file 1 [file marinedrugs-21-00490-s001.zip › marinedrugs-2600326-supplementary.pdf]

Supporting Information

Activation of a Silent Polyketide Synthase SlPKS4 Encoding the C_7-methylated Isocoumarin in a Marine-derived Fungus *Simplicillium lamellicola* HDN13-430

Jing Yu [1,†], Xiaolin Liu [1,†], Chuanteng Ma [1], Chen Li [1], Yuhan Zhang [2], Qian Che [1], Guojian Zhang [1,3], Tianjiao Zhu [1,*] and Dehai Li [1,3,*]

[1] Key Laboratory of Marine Drugs, Chinese Ministry of Education, School of Medicine and Pharmacy, Ocean University of China, Qingdao 266003, China; jingjingyu95@163.com (J.Y.); 17865327317@163.com (X.L.); ma_chuanteng@163.com (C.M.); 17629508296@163.com (C.L.); cheqian064@ouc.edu.cn (Q.C.); zhangguojian@ouc.edu.cn (G.Z.)
[2] School of Pharmaceutical Science, Shandong University, Jinan 250100, China; 202100260059@mail.sdu.edu.cn
[3] Laboratory for Marine Drugs and Bioproducts, Pilot National Laboratory for Marine Science and Technology (Qingdao), Qingdao 266237, China
* Correspondence: zhutj@ouc.edu.cn (T.Z.); dehaili@ouc.edu.cn (D.L.)
† These authors contributed equally to this work.

Table of Contents

Table S1. The primers used in this study. (5' to 3')....2
Table S2. Analysis of gene cluster....4
Table S3. NMR data of **1** in DMSO-d_6. 5
Table S4. NMR data of **2** in CD_3OD....5
Figure S1. RT-PCR analysis for gene transcription status. The results showed that all seven tailoring enzymes together with SlPKS4 were totally silent under 6 regular laboratory conditions. 6
Figure S2. AntiSMASH analysis results of the genome of the strain *S. lamellicola* HDN13-430....7
Figure S3. UV absorptions of compounds **1** and **2**....7
Figure S4. Maps of the vectors pANU-*SlPKS4*, pANU-*SlPKS4+Sl4004*, pANR-*Sl4001+5+7* and pANP-*Sl4002+3+6*....8
Figure S5. HPLC full chromatogram of the original *A. nidulans* and the strain harboring SlPKS4. The results prevent the presence of compound **2** in original *A.nidulans*....8
Figure S6. RT-PCR analysis for gene transcription status. The results showed that all seven tailoring enzymes together with SlPKS4 were expressed properly. 9
Figure S7. Comparative analysis between the gene cluster and other isocoumarin BGCs by clinker. 9
Figure S8. ^1H NMR (500 MHz, DMSO-d_6) spectrum of compound **1**. 10
Figure S9. ^{13}C NMR (125 MHz, DMSO-d_6) spectrum of compound **1**. 10
Figure S10. ^1H NMR (500 MHz, CD_3OD) spectrum of compound **2**....11
Figure S11. ^{13}C NMR (125 MHz, CD_3OD) spectrum of compound **2**....11
Gene Sequence of *SlPKS4* 12

Table S1. The primers used in this study. (5' to 3')

Primers	Sequences
Primers for pANU-*SlPKS4*	
SlPKS4-F1	CTGAGCTTCATCCCCAGCATCATTACACCTCAGCAATGGACAG CTCTACTCCTTCTTTC
SlPKS4-R1	GCAATACACCCGTCTTTAGCTGC
SlPKS4-F2	GTCCTTACCAAGGTTTGAAGAGTGC
SlPKS4-R2	GTGGAGGACATACCCGTAATTTTCTGGGCATTTAAATCCAGAT ATTCCACGCGTGTCATG
YZ-pANU-F	ATATGAGTTCATCCTGCAGAATACCG
YZ-SlPKS4-R1	GATATGATTTCCGTTGGTGATGGTG
YZ-SlPKS4-F2	AGTATGAGAATCTACGCCTACCTCC
YZ-pANU-R	CTTACCCGAGTACACATAGCCGTAC
Primers for pANU-*SlPKS4+Sl4004*	
Sl4004-F	GACTAACCATTACCCCGCCACATAGACACATCTAAACAATGAC AAGATCGCACCGAGTC
Sl4004-R	ACAGTGGAGGACATACCCGTAATTTTCTGCGTAAGAGAGAAT AGAGTAGAAGCGACAAG
YZ-Sl4004-F1	TTGGTCGTCTGTGATGGAAGGATAC
YZ-Sl4004-R1	GGTACTCACTCCTACGATCTCGAG
YZ-Sl4004-F2	CAATTCGCACCAATCACAGCAC
YZ-pANU-R	CTTACCCGAGTACACATAGCCGTAC
Primers for pANR-*Sl4001+5+7*	
Sl4001-F	TAACCATTACCCCGCCACATAGACACATCTAAACAATGGCCAC AATGAGAGCTGTTG
Sl4001-R	GACCAGTTCGGAAGATCAGGGTTTAAACGCGGCCGCGCACGG AGAGGGAATCTATGCTG
Sl4005-F	GAGCTTCATCCCCAGCATCATTACACCTCAGCAATGTTGTCTC GTTGCGCTGTTC

Sl4005-R	CTCGTTCGGCACCTTTAATCTTAATTAAGTTTAAACGATAGCC AGGCACCAGATTCCAG
Sl4007-F	CTCTGAACAATAAACCCCACAGAAGGCATTTATGGCCTCAAG AACTTTCTCCAAG
Sl4007-R	GGGTATCATCGAAAGGGAGTCATCCATTAATTAAGATGCTTCG ACGCTGTTCTAGAACC
YZ-pANR-F	CTCGAGATCGTAGGAGTGAGTACC
YZ-Sl4001-R	GAACCAGGTGAAGAGCCTGTGTAG
YZ-pANU-F	ATATGAGTTCATCCTGCAGAATACCG
YZ-Sl4005-R	GTAGTAGTGGTCTTGAGCAGGGTC
YZ-Sl4007-F	TAACATTACCTCTTCCACAGTGCCC
YZ-pANR-R	GACGGACTTGAGAGGGAGGAG
Primers for pANP-*Sl4002+3+6*	
Sl4002-F	CCCTTCTCTGAACAATAAACCCCACAGAAGGCATTTATGGCCG ACGAAAACGCC
Sl4002-R	AGTTCGGAAGATCAGGGTTTAAACGCGGCCGCGCGTCGACTTT ATTGTTGACTTTGTCG
Sl4003-F	AGCTTCATCCCCAGCATCATTACACCTCAGCAATGGGTCTTAC AGATGCTTCCCC
Sl4003-R	ACCCAAATCAATTCACCGGAGTTTAATTAAGTTTAAACACGAC GTCAAGTTCAAGGCTG
Sl4006-F	CATTACCCCGCCACATAGACACATCTAAACAATGACAGACTTC AAAGCATTGGCC
Sl4006-R	GGAGTGATGAGACCCAACAACCATGATACCAGGGGACATTCT ATCCTCTCGGCCTGTTG
YZ-pANP-F	CTCGCTTACCGATTACGTTAGGG
YZ-Sl4002-R	GATAGAGGTCGAGTCTTCGGCAATG
YZ-pANU-F	ATATGAGTTCATCCTGCAGAATACCG
YZ-Sl4003-R	ACGAGACTCGCTCCATGATAAGG

YZ-Sl4006-F	GTATGTGGTTACGTATCATTCAACGC
YZ-pANP-R	GTACCGTTGGAAGCCATTCTGTG
Primers for amplification of promotors	
pANP-F	GATTAAAGGTGCCGAACGAGCTATAAATG
pANP-R	AAATGCCTTCTGTGGGGTTTATTGTTC
pANU-F	CCTGATCTTCCGAACTGGTCGTAC
pANU-R	TGCTGAGGTGTAATGATGCTGGG
pANR-F	ACTCCGGTGAATTGATTTGGGTG
pANR-R	TGTTTAGATGTGTCTATGTGGCGGG

Table S2. Analysis of gene cluster.

Gene name	Length (bp)	Predicted function	Similarity/Identity (%)	Genebank accession number of most similar enzyme
SlPKS4	7674	Polyketide synthase	99/56.38	KAI9661722.1
Sl4001	1088	quinone oxidoreductase	99/81.02	PNY24818.1
Sl4002	1128	dienelactone hydrolase	100/73.44	OAA34895.1
Sl4003	1501	γ-glutamyl phosphate reductase	47/78.92	XP_011319059.1
Sl4004	1574	hypothetical protein	85/24.61	KAE9373914.1
Sl4005	1347	sulfide quinone reductase	100/67.71	OAA75573.1
Sl4006	1147	threonine dehydratase	99/68.34	OAA77147.1
Sl4007	1439	ketol-acid reductoisomerase	100/89.83	KAF5127587.1

Table S3. NMR data of **1** in DMSO-d_6.

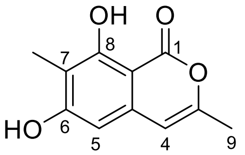

1

No.	δ_H (J in Hz)	δ_C
1	-	166.54
3	-	153.63
4	6.45 s	104.64
4a	-	136.88
5	6.39 s	101.94
6	-	164.35
7	-	110.10
8	-	160.37
8a	-	97.83
9	2.19 s	19.23
7-CH_3	2.00 s	8.40
8-OH	11.27 s	-

Table S4. NMR data of **2** in CD_3OD.

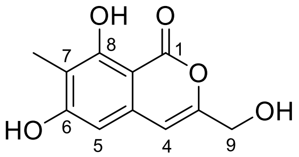

2

No.	δ_H (J in Hz)	δ_C
1	-	166.48
3	-	155.00
4	6.47 s	103.55
4a	-	136.11
5	6.36 s	102.32

6	-	165.27
7	-	111.12
8	-	160.59
8a	-	97.64
9	4.34 s	60.01
7-CH_3	2.09 s	6.64

Figure S1. RT-PCR analysis for gene transcription status. The results showed that all seven tailoring enzymes together with SlPKS4 were totally silent under 6 regular laboratory conditions.

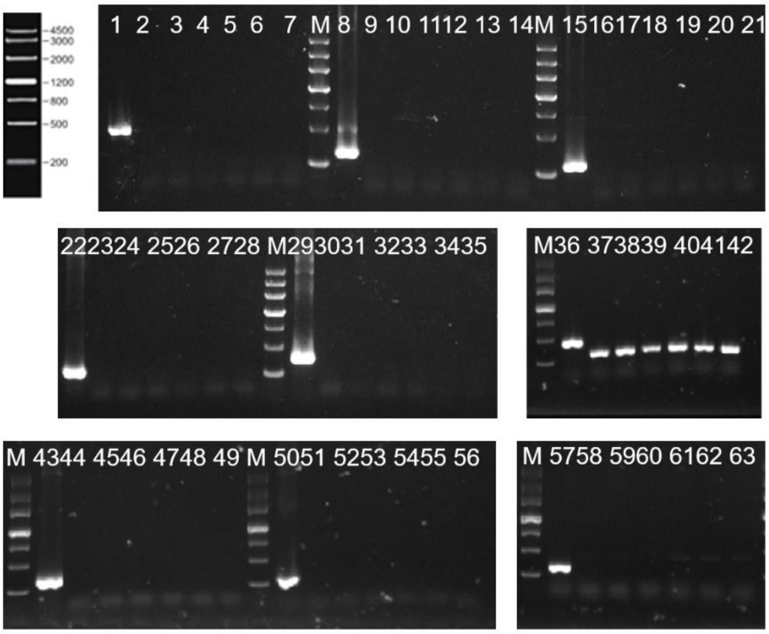

No.	Gene-gDNA	Length (bp)	No.	Gene-cDNA	Medium respectively
1	SlPKS4	415	2-7	SlPKS4	1-6
8	Sl4001	293	9-14	Sl4001	1-6
15	Sl4002	238	16-21	Sl4002	1-6
22	Sl4003	234	23-28	Sl4003	1-6
29	Sl4006	313	30-35	Sl4006	1-6
36	tubulin	-	37-42	tubulin	1-6
43	Sl4004	320	44-49	Sl4004	1-6
50	Sl4005	287	51-56	Sl4005	1-6
57	Sl4007	340	58-63	Sl4007	1-6
M	Marker III (Tiangen biotech, Beijing)				

Figure S2. AntiSMASH analysis results of the genome of the strain *S. lamellicola* HDN13-430.

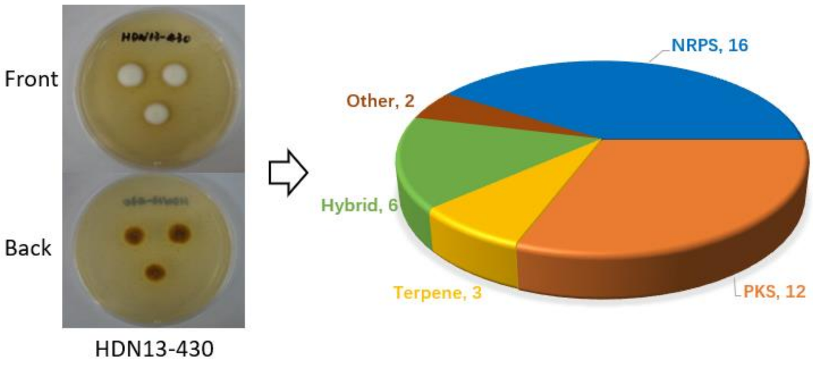

Figure S3. UV absorptions of compounds **1** and **2**.

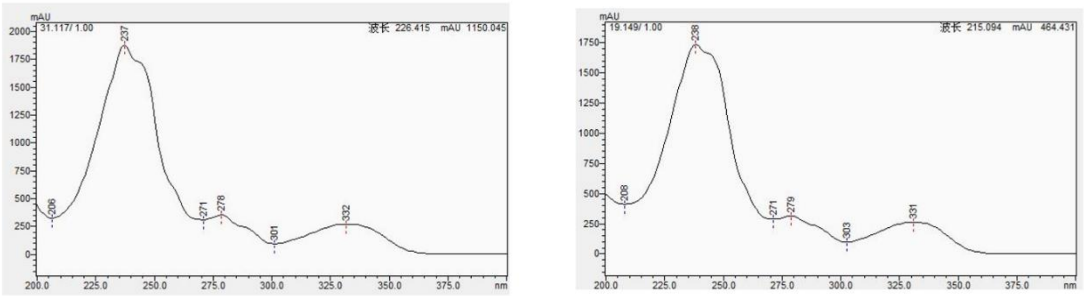

1 **2**

Figure S4. Maps of the vectors pANU-*SlPKS4*, pANU-*SlPKS4*+*Sl4004*, pANR-*Sl4001+5+7* and pANP-*Sl4002+3+6*.

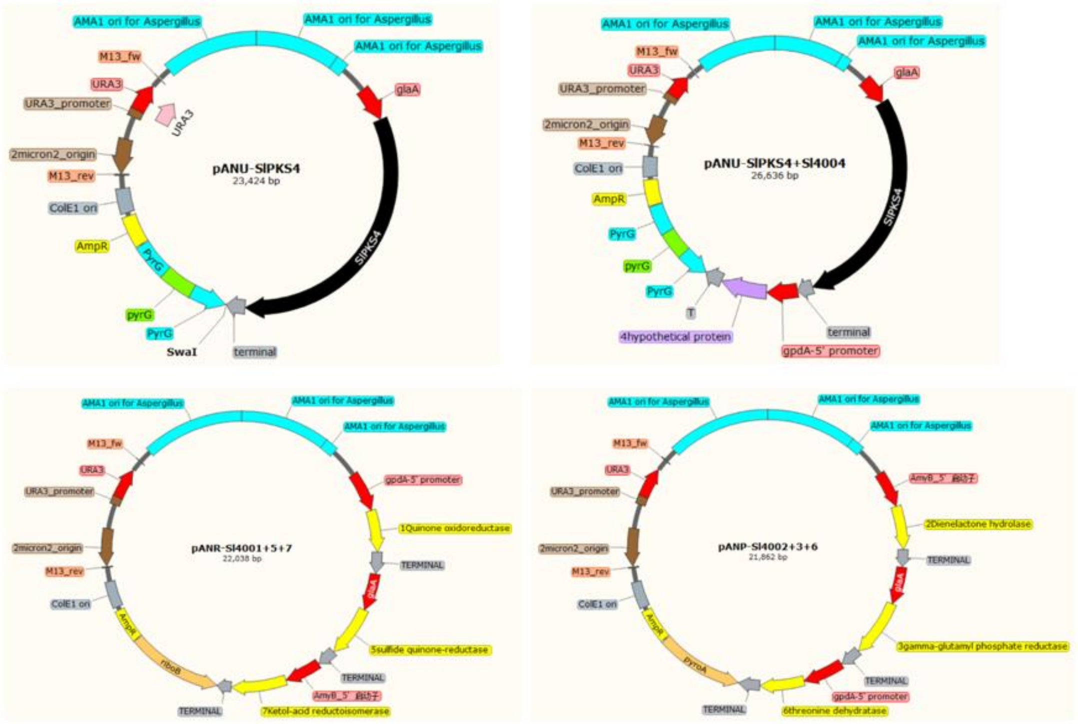

Figure S5. HPLC full chromatogram of the original *A. nidulans* and the strain harboring SlPKS4. The results prevent the presence of compound **2** in original *A.nidulans.*

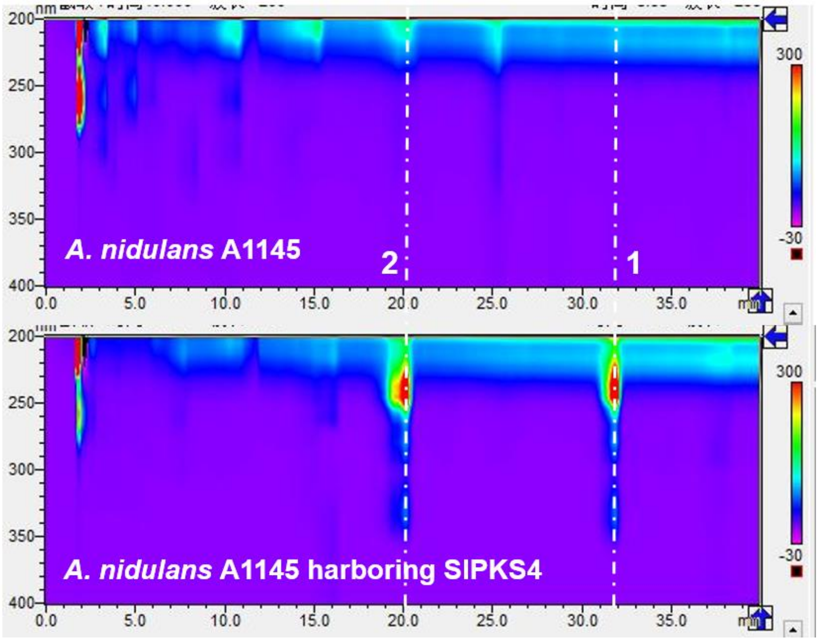

Figure S6. RT-PCR analysis for gene transcription status. The results showed that all seven tailoring enzymes together with SlPKS4 were expressed properly.

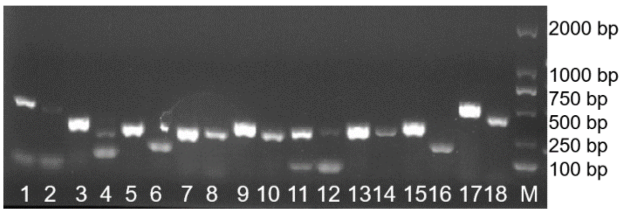

No.	Gene-gDNA	Length (bp)	No.	Gene-cDNA	Length (bp)
1	SlPKS4	415	2	SlPKS4	359
3	Sl4001	293	4	Sl4001	206
5	Sl4002	238	6	Sl4002	124
7	Sl4003	234	8	Sl4003	221
9	Sl4004	320	10	Sl4004	263
11	Sl4005	287	12	Sl4005	287
13	Sl4006	313	14	Sl4006	260
15	Sl4007	340	16	Sl4007	181
17	tubulin	-	18	tubulin	-
M	Marker D2000 (Tiangen biotech, Beijing)				

Figure S7. Comparative analysis between the gene cluster and other isocoumarin BGCs by clinker.

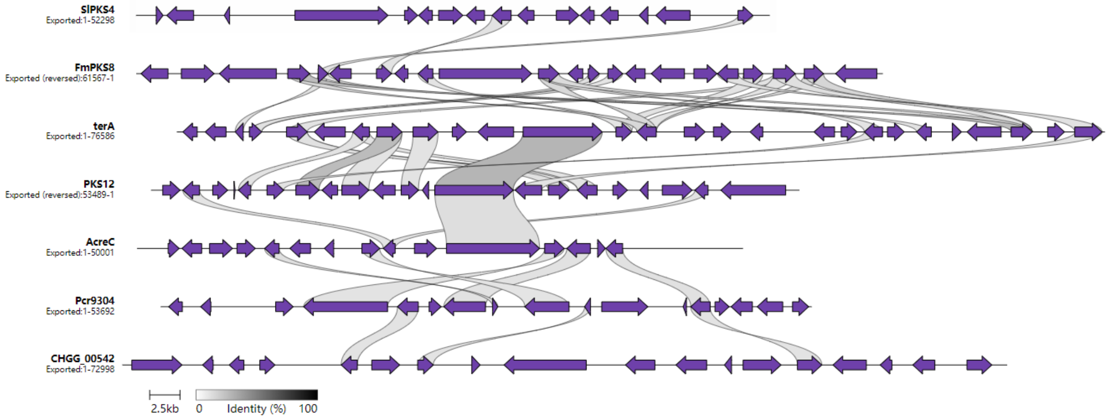

Figure S8. ^{1}H NMR (500 MHz, DMSO-d_6) spectrum of compound **1**.

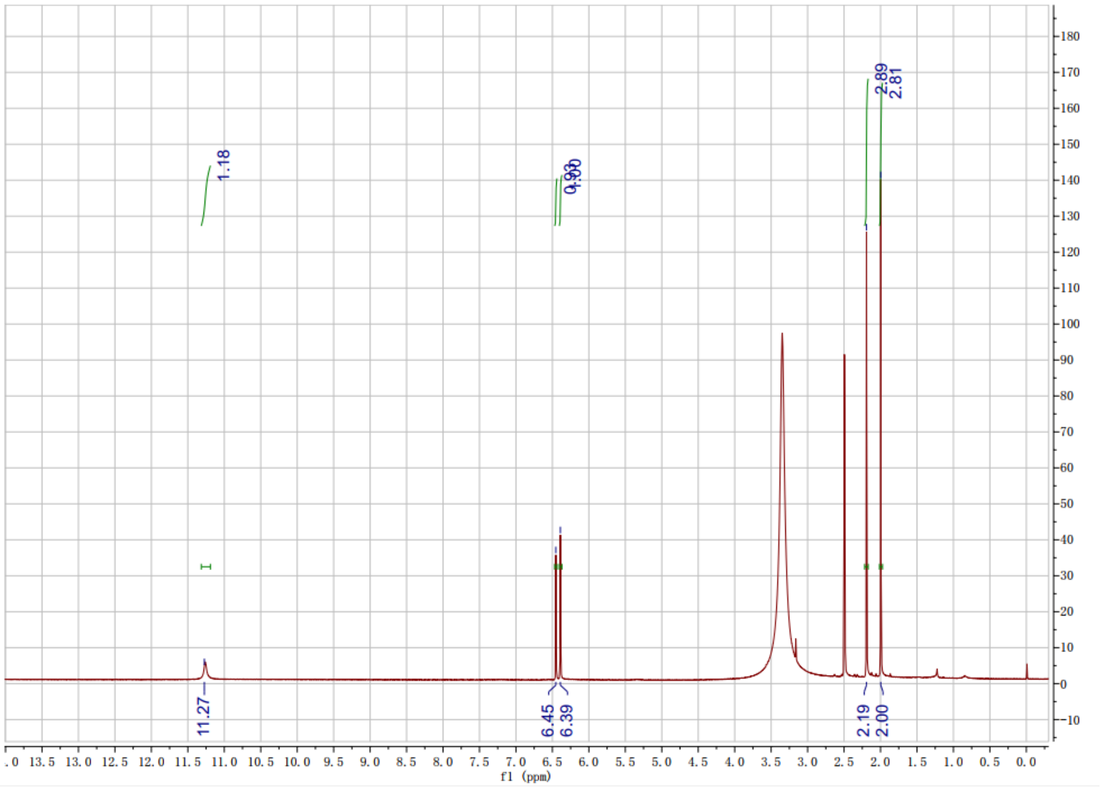

Figure S9. ^{13}C NMR (125 MHz, DMSO-d_6) spectrum of compound **1**.

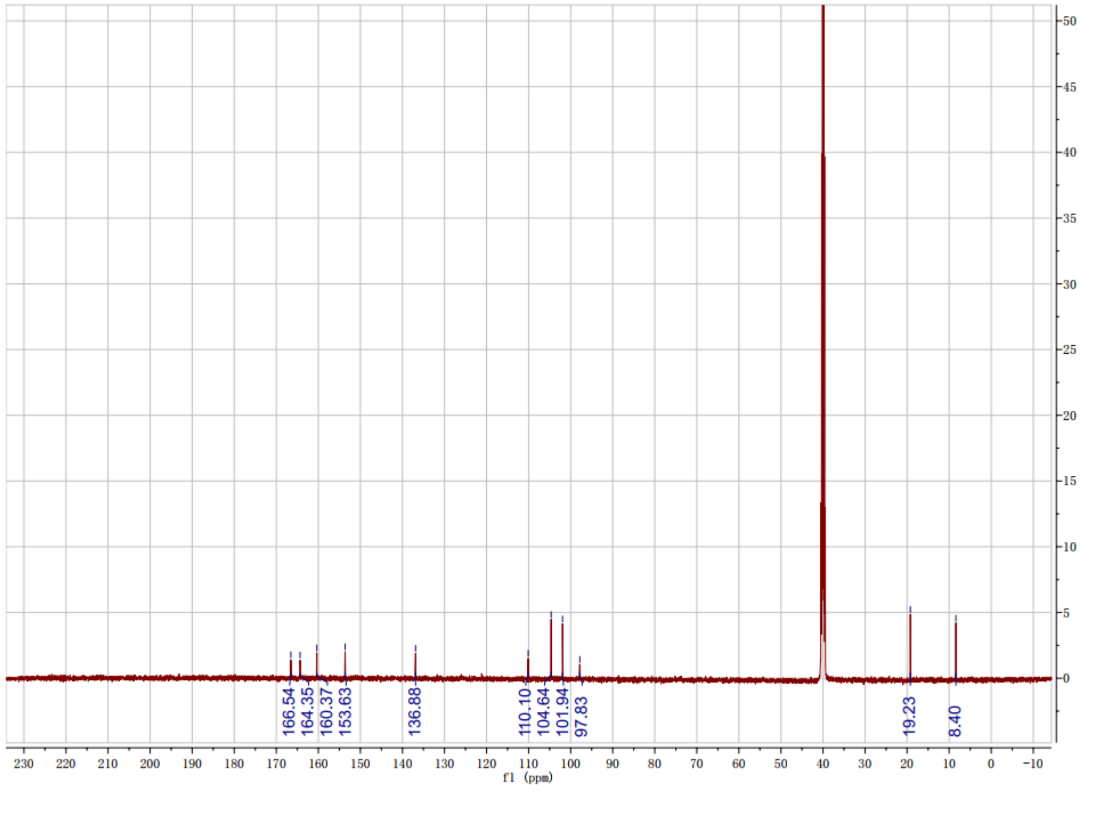

Figure S10. ^{1}H NMR (500 MHz, CD_3OD) spectrum of compound **2**.

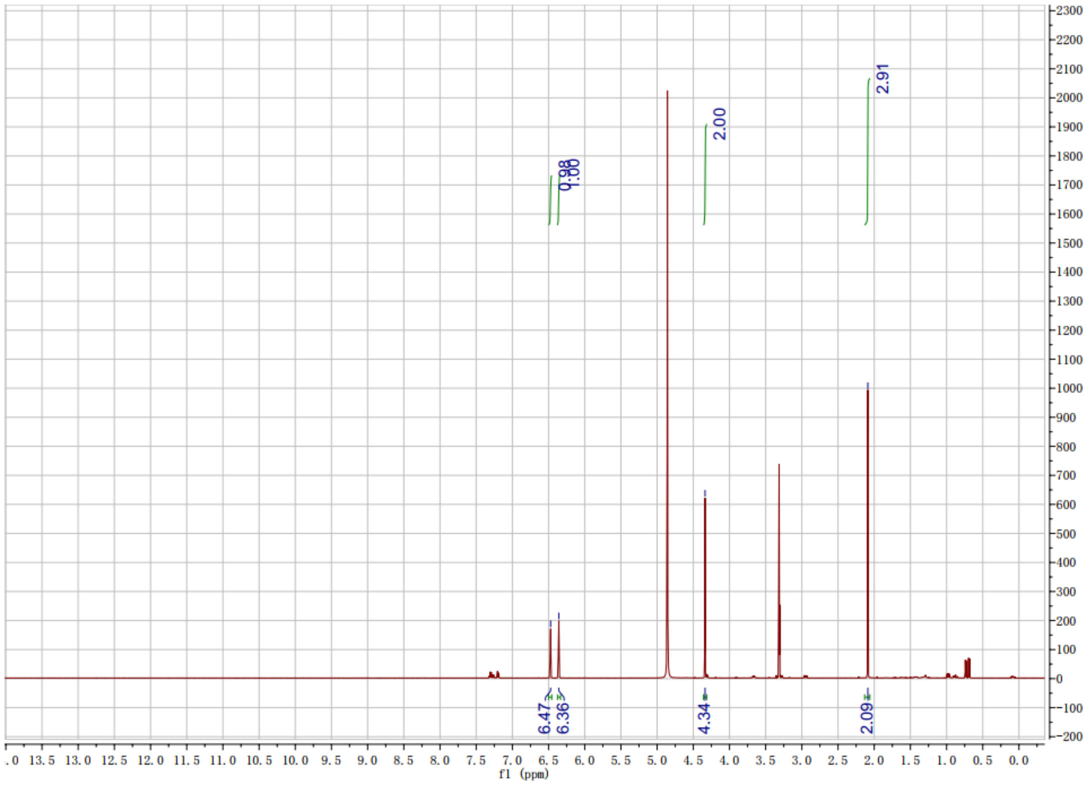

Figure S11. ^{13}C NMR (125 MHz, CD_3OD) spectrum of compound **2**.

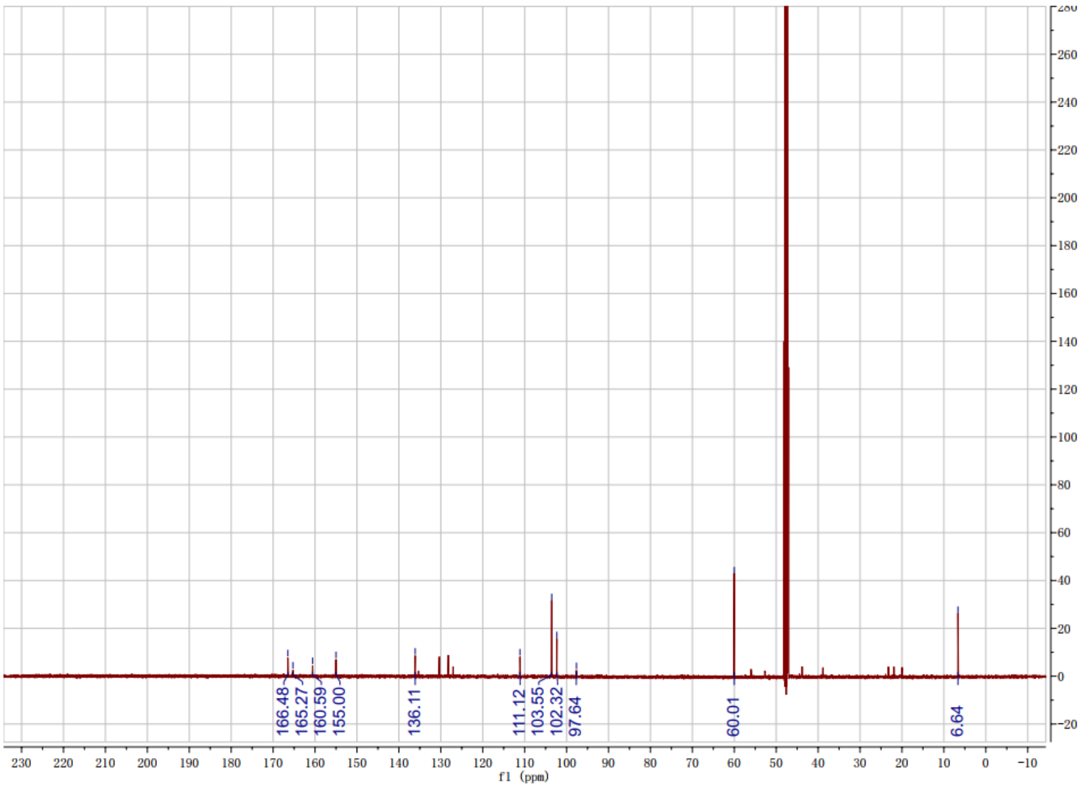

Gene Sequence of *SlPKS4*

>*SlPKS4*

ATGGACAGCTCTACTCCTTCTTTCATTGCCTTTGGCTCACTGACGCCGTGGC
CAACGCCAGCAGAGCTCACCCAGCTAAGACAAGCGTTGCTACAGGTCCAA
GAGCTGCAGCCAATGTGCGAAGCATTGAAAGAACTGCCCAAACTATGGGA
TACATTGTCCAATCATGATTCATCCCTGCAAAAAGTAGAGGGTGCCTTGGCT
GCTGAGCAGCTGAGCCAGTGGCTGTTGACCGGTCAAGCACCTACTCGAGA
TTTCGCAAGAAACAATATCCTCAGCTTACCATTAACCATCCTTTCTCATATTT
CCGATTACTATGTTAACTTTCATCAAAAAGAGTCAAAACTTGACCGCGCCA
AAGTCGAGGGCGTCTGTGCTGGACTTTTGAGTGCCTTGGCTGTCCGCTCTG
CTGAAGGCTACAGTCAGGTAGGCTCACACGGGGCATTGTCCATCTTGTTGG
CGTTTTCCGTTGGCACATATGTTGACATGGACGCTTTACAACATGGTCAAAC
GACGTGTCTTGCTGTGAGATGCAAAGCCCCTGCTACTCTTGACACCGTCCG
AGATACATTAAAAGATTATGATTCAGTATGTCATACGTGCAAAACATTGTGT
GAATAATCGCCCCCACTAACATTACCAGGCATATCTTTCAGTTATAAGAAAC
GCAAATGAAGCAACCATAACAGTTCCTTCATTTTGTGCTGCACAAATATCGC
GACGATTATCTGCTGATGGCATTAGCGTGATGGATACCGGACTTACAGGCCG
TTACCACTCTCAGTCTCACTCATTAGCACCTGAACAAATCAGCTCTGCCTTT
ACAGAGCTGGGGTATAATTTGACAAATGACGCTAAGAAGGCAGTCCACGG
CATCTTGGTTGAGGTTTCCGACTGGTCAGCCCAATTTGAAGCTGCAAACAT
TTCGCAAAACGACGTCGTCCTCACTATCGGAGACGCTGTCAAGTGCTCATT
ACCTCGCAACACCAAGGCCATTGGAAACGACAAAGTCAAACAATCTACCG
AGGATCTTTTGGCCGATTATCCTGCAAATGCCATCGCCGTGGTGGGCATGTC
TTGCAAACTGCCTGGAGCAGATTCCGTCGAAGAGTTCTGGCAACTTCTTAC
AAGTGGAAAGTCTATGGTAGAACAGGTCCCTGAAGATCGATGGCCTGAATT
AGCAGCGACTCGAGGAAACTCAAAAACAAAAAAGTACTGGGGCAACTTC
TTAAAAGACATTGATGCGTTTGACCATCGATTTTTCAAAAAGTCCGCCAGA
GAAGCCGCTAGCATGGATCCCCAACAGCGACTTCTGCTGCAAGCCGCCTAC
GAAGTCCTGGAATCGTCGGGATACTATCAACCATCACCAAACTCCCCTTCTT
CATCTACCAAAGCGCGAGATATCGGATGTTACATTGGTCTTTGCGCAGTCGA

CTACGATATGAACACGACATGCCATGCGCCAAATGCCTTTTCTACATTAGGA

ACACTAAGAGCTTTCCTGAGTGGCAAACTTTCTCATTTCTTTGGCTGGTCA

GGACCTTCATTGACGTTCGACACGGCATGTTCCTCATCTGCTGTAGCAATCC

ATACAGCTTGCCGGGCCCTGCAAGCCGGCGAATGTTCACAGGCGCTCGCTG

GTGGAGTGGCCTTATTTACCAGTCCTTACCTGTATGAGAACCTCGCTGCCGC

ACACTTCCTCAGCCCAACTGGAGCGACCAAGCCATTCGATTCCAAGGCAG

ATGGATACTGCCGAGGTGAAGGATTGGGCCTTGTCATGTTGAAGAAGTTGT

CGAATGCAGTTGCAGATGGTGACGATGTTCTAGCTGTGATTGGAGGGTCTG

CCATCAACCAAAACGATAGCTGTGTCCCAATTACAGTCCCAAATGCTCCAT

CTCAGCAGAATCTATACCAGAACGCTGCCAAACAAGCCGGTATTAAGCCAC

AGCAAGTATCGTTTGTCGAGGCTCACGGTACTGGAACGCCAGTGGGAGAT

CCAATTGAGATGGACAGCATTCGTAATGTCTTTGGAGGCTCCAGTCGCCGC

AATCAGCTGGTTGTGTCCTCCGTCAAAGGAAACATTGGTCATCTTGAGGGC

GCATCTGGAGTGGCTGGTCTCATTAAGGCTATTCTTCAAATTCAACACCGCA

CCGCAGTCATCCAAGCTTCATTCCAATCTCTCAACCCCAAAATCCCAGCCTT

GGCACCCGATAACATTGTCATTCCCACCACCAACGTTCCGCTTACTGACCC

ATTCTTGACTGCATGTGTCAACAACTATGGTGCTGCTGGAAGCAACAGCAC

GTTGATGGTCATGCAGCCACCGAAAACGAATTCTATAGAAGTCTCGCATCT

CTCAAAGTACCCCATCGCCATTGCTGCCAACTCCGAAGCTGCCGTTCAAGA

GTATTGTCGTGCGTTACAGGATTATGCTTTCAAGAACCCTCAGAAAATGCA

GCAGATGGCCAGTATTGCCTATCATCTCAGTCGACGACATAACCAGAAGCT

CCCATATCTCATTACCGCCACTGCATCAAATCTACAAACTGCATTCACACAA

CCCGTCGCGCAACGGAAATCTCCTATATCTCTCGTATTGGCTTTTGGTGGCC

AAGTGAGAGATAGTGTCGGCTTGAGCAAAGAGATCTGGAATCAGTTCGCT

ATTCTTCGTCTGCATCTTGATCGTTGCGATGAGATTTTGCGATCAATGGGCC

ATGCGTCGTTGTATCCGGCCATCTTTCAATCAGAGCCTATCCATGAACTGGT

GACTCTTCATTCTGCTGTTTTCGCAATACAGTACGCCTCGGCTAAGTCATGG

ATTGAATGTGGCCTTAAAGTCGATTGTGTCATTGGTCACAGCCTGGGACAG

CTCACAGCATTGACTGTCTCTGGTATACTCTCATTGCAAGATGGGTTGAAGT

TTGTGGCTGGACGAGCTACGCTTATGAACAAGTACTGGGGTCCTGAACCTG

GCTCCATGATTCTGGTAGATGCCAACGCTCAGTCAATAGCAAAGTTGCCAC

ATTCTCTTGAAACGGCCTGCTACAACGGACCTGCAGCTCAAGTGATGGTGG

GCGAGCAGGCGGCCGTCGACAGCTTTGAAGATGTTTTGACTCAGAACGGT

ATCCGATTCAAGCGACTTCAAGTCGCCAATGGGTTCCATTCCAAATTCACA

GATCCTCTTATTGAGCCACTTACGAAATTGGCCGCTACGCTTCGTTTCAATA

AGCCTGTGATCCCTATCGAGACTTGCTCTCTTGGTGAATCCTGGGCTCAAGT

GACTCCCGAGTTACTTGCCAATCACACTCGTGAGCCAGTCTACTTTCACAA

AGCTGCCCAACGAATCTCTAGTCGTGGAGTATGCACCTGGCTAGAAGTTGG

CTCAGATTCAGGAATCACAGGCATGGCTAAACGCGCCTTGACTACCACTGA

TGACGAATATCTTGGAGCAACACTCAGCAAATCGTCAGCCTTGGACGCAAT

TATCGATAATACCACCAAGTTATGGAAATCTGGCCATGCCACGCAGTTTTGG

GCTTTCCATCACTCTCAGAGGAGCCAGTATGAGAATCTACGCCTACCTCCGT

ATCAATGGGAGAAGAACAGACATTGGCTTGATTTACTTCCGCCAAAACAGC

TTGAACTGGCACCTGCAAACGCACCTGTCATTGAAAATGAAGTGCCACAG

CTCGTCAAACTTGTGTCCCAAAACTCATCAGAGGCGGTTTTCCGTATTGGA

GTTCAATCTGAAGAGTATCAGCGCTTCGTGTCTGGCCATGTAGTTGCTGAC

AGTCCTCTTTGCCCAGCAACAGTATACATGGAAGTATCTGCTAGAGCATGC

AAAATGATTTGCACTTGGGAGCCCGCACCGCACCTTGGATTCTCTGATTTG

AAGATTGACTCGCCGCTGGGAATGTCTGCAAGCAACGAGATTACAATGACC

CTCCGCCAATTATCCAAGGATGATTGGGAGTTCAACGTTATGAGCTCTAAAG

AGTCGAACAAGAAGGTATCTCATGCGACGGGAAAAATTCATCTCAAACAA

GACGTCGCTGCTGTACAGAGAGATTTGTCACGATACGCTCGACTCGTTCGC

CCCAGTATGGTTCAAGCTCTGTTGGATGACTACAACAGCGAATGCGTCCGC

GGATCCATGCTCTACAAGCTATTCTCTAGAGTAGTACAATACAGTGGTCCTT

ACCAAGGTTTGAAGAGTGCTGCAGCTAAAGACGGGTGTATTGCTGGAGTG

GTTACTGCAGATGCAGACGATACCGAAGGCACTGTGCTTTCTCAGCCTCCT

ACTATCGATAGCTGGATGCAAATCGCTGGTTTCCACTCCAACAACTTCTATC

CATGCTCAGAGGATGATGTGTACGTCTTCACGAAGGTGGACAATATGCAAT

TCGGGCCCGACTTCGACCAATCCAATACAAAGTCATGGAAGATTTACTCCA

ACTTGACACCAATCGAGAACAATGAGCTATCCAACGATATTTCGTCTTTGA

TTCAGTTTCAGACAAGCTAGTAGTACTTATCCTTGGCGCTCGCTTCAACAA

GGTGAAGCTAAATTCACTCAGTAAAGTACTATCGAGACTCAATGGCACTGT

CCCCACCACCAAGAAGGAACCAGTTCCTATGAAGATGGAACGTCAGCTTC

CACTTACACCATCACCAACGGAAATCATATCCAAGATGAGATGCGGCGCTC

GCGAAACTATTCTTGAAGGTATCAGCTCTGTATTTGAGCTGGTAGCAGAAG

TACCGCGTGAAGAAATCAAGGGCAGCGCAACCGTCGATGACTTGGGTATT

GATTCTCTCATGATGATGGAGGTAGTCAGTGAACTATCAAGTTACTTCTCTA

TCGACCTTCCAGTCGAAGATATGGAGGGATTGACTGATGTCGACTCCCTCA

CTGGTTATCTACTGAAACGTGGATGTGGCCAAGACTCAGCAGCGTCTTCGA

CAATCTACTCCAGTAGCTCATCCGTCAGTCCTGGAACGCCATTGACCAGCG

CCTCTTCAAGCAATGAAGAGCAAGTTGATCAACAGGTTGAGCAGCTCGCC

AAGTTGCTTCAAGAGCATCTGGAGTTGGACACTCTGCCGAATATGTGTGAT

AACCTTGGGGATTTGGGATTGGATTCGCTTCTTGCAATTGAGCTTGGCGAC

GACATTGAGAAGATGCTCTCTGTGACCATTGATTTACACCAGATTGATGAA

AAATCTACTGTTGGCGATCTTGTCCAACTTGCTGGGCTGAATGCTGAAGCC

AACTTCACGTCGTCAATCCCACAAGTAACACTCTCCGAGCCAATGATGCCT

GTCACTGTTGCCCCAACAGAGTCAGTGAAGTCCGTAACACCTTCAAATACC

AGAGATATTCACGAAGCATTCGATGAAGTCCGTTTAGATTTCGATGCATTCG

CAGAACAAGAAGGATTCACAAACTTTTGGAGCACAGTCTACCCAGCTCAA

GAAGAACTTGTCCTTTCATACGTTGCCGACGCCTTTCGAAAACTCGGATCT

GATATCTCGACTTTAAACAGCGGAGATAGCATCCCTTCACTATCAGTTCTCG

ACAAGCATCAACATTTGGTAAAGCGTATGCACAAGATCTTAATGGACGGTG

GCTATGTCTCCGAACAAGACTCCAAGTACATCCGCACCGCAAAGAGATTTA

ACTTGGCCCTCCCACAAGTTTTGCTCAGCCGCATTATATCACAGTTTCCCCT

TCACGCCTCTGAACACAAACTACTTGATATCACTGGATCGACGTTGGCAGA

GTGCCTTACTGGAAAAGCTGACCCATTAACGCTCTTGTTCGCAAAGAAGTC

GAACCGCAAGCTTCTCGCCGATGTCTATGACCTTGCACCTATGTGTCGCGCT

GCCACTCGATTGCTGGGCAAGTTTCTCGAAAACGCCTACGCTAGCAATGCC

AATGGACAAACCTACCATTTCTTGGAAGTTGGTGGCGGAACTGGTGGTACA

ACCAAGTTCGTAGTGGAATATCTCACGCGCAAGGGAGTTGACTTCACTTAC

ACGTTTACGGACATCTCTTCTGCTCTTGTTGGTGCGATGAAGAAGGAAATG

TCTGAATACGACTGTATGCGATATACGACACTTGACGTGGATAAGCCAAGCT

CAGAATTGACCGGACAGTACCACGCCGTGATTTCTACCAATTGCGTCCATG

CTACTGGTAACGCCACTGCTGCTTTGGCCAATCTTCGTACATTGCTTCGTCC

AGATGGTATCATGGCCCTAGTGGAGTTCACTACTGGACTGTACTGGTTCGA

CCTGGTGTACGGCCTTCTCGATGGTTGGTGGCTGTTTAGCGATGGTAGAAA

GCACGCACTGGCGGATGTGCCTTTCTGGGAAAAGAGTATGCTGGCCGCGG

GCTATCAGCACGTCAACTGGTCAGATGGTGATACTCGGGAGTCTCGAACAC

TAAGACTTATCTGCGGGTTCAATTCTACCCCATCGTTTTCCAGTCAGCCAAC

TGCACCTATTCAGAAGCGAGCCGGTGTACAATACGAGACTGTCACATGGAA

GCAAGTTGGCAGACTTGACCTGAATGCCGACATATACTACCCTTCTGAACG

GGATACGAACACCAAAAGGCCTGTGGGTAAGTAAACAGCAGCCCACTAGG

ATCTGAATACCCGCTGACCATATTCAGCTGTGTTGTTCCATGGGGGCGGACA

CGTCTTATTCACGCGAAAAGATATCCATATTAAGCATGTCAAGATTCTGCTG

GAACGAGGATTCCTCCCCATCAGCTTTGACTATAGATTGTGTCCCGAAGTG

ACACTTTCTGAAGGCCCAATGACAGATGCATGCGATGCATTCCAATGGGCT

CGTGAGCAGCTTTCTCATCTGCGTCTGGCACGACCTGATATTCAGATCGAC

GAGAACAGAGTTGTTGCTGCTGGATGGTCTGCTGGTGGTCAACTGGCCATG

ACACTTGGATACACTGCGCCAGCTAGAGGATTGAAAGCTCCTGATGCAGTG

CTGGCATTCTACTGCCCGACGAATTTCGAAGATAGCTGTAAGTATGCGACA

CTGCATAGGACTTGAACGGATGCTAATTCAGTAGGGTGGACGAAACCAATT

TATCCTCAGGGGATTAAAGAGGAACCAGGCACAGACTATGACTTACTAGAA

GGAGTTTTGGACAAACCGGTAAGTGACTCAACAGACAAGACTATGACAGA

TATCAAGCTAACTTCATCCAACAGATGACTGGATATAAACCACCTACCCTTC

CAGGAGCACCGATGAGCCTACAAGACCCACGATGGCGTATCATCATTCACT

ATAACTGGAAAGCACAATTGGTACCTGTTCTAGTCAATGGCCTCCCATCGA

AGGGCAAGTCAGGATGTCACAACTTGAAGGATTTGCCCATGCCGCCCACG

GAGAAGATTCAAGCCGTGAGTCCATTCGCCCAAATTGTCAAGGGCAAATAC

CACACTCCAACATTCATCATTCATGGTGAATTGGACGATTTGATCCCCTGGC

AGCAAAGCCGAGATACAATCGACGCGCTTAAGATGCAAGGAGTGAAAGCT

GGACTGGCAACTCCCAACGCTGGCCATGCATTTGATCTATGGGGTGACAAA
ACGGATTGGTCGTCTGTGATGGAAGGATACGACTTTTTGCAGTCTCATTCTT
GA
